# Rapid forensic ancestry inference in selected Northeast Asian populations: a Y-STR based attention-based ensemble framework for initial investigation guidance

**DOI:** 10.3389/fgene.2025.1631529

**Published:** 2025-09-17

**Authors:** Kyo-Chan Koo

**Affiliations:** Department of Management Engineering, College of Science and Technology, Dankook University, Cheonan, Republic of Korea

**Keywords:** Y-STR, rapid forensic screening, initial ancestry inference, machine learning, data imbalance, Northeast Asian populations, crime scene investigation

## Abstract

**Introduction:**

Rapid inference of ancestral origin fromDNA evidence is critical in time-sensitive forensic investigations, particularly during the initial hours when crucial investigative decisions must be made. Although comprehensive analyses using multiple genetic markers provide thorough results, they often require significant processing time and resources. Y-chromosome short tandem repeats (Y-STRs) exhibit population-specific allelic distributions that facilitate rapid analysis, making them particularly valuable for initial screening in forensic contexts.

**Methods:**

This study aims to enhance population classification accuracy using Y-STR profile analysis, with a particular focus on Northeast Asian populations that are often merged into a single group by commercial ancestry panels. We developed a machine learning architecture centered on an attention-based ensemble mechanism that incorporates three complementary algorithms: a One-vs-Rest Random Forest, XGBoost, and Logistic Regression, each configured to effectively manage imbalanced datasets.

**Results:**

Utilizing only Y-STR data, the model achieved an overall accuracy of 80%-81% and demonstrated high stability. Notably, the model effectively processes imbalanced datasets, generating reliable outcomes for rapid ancestry assessment in time-critical investigations.

**Discussion:**

By addressing a key limitation in commercial ancestry panels--their failure to differentiate among Northeast Asian subpopulations--this framework provides valuable preliminary guidance in forensic cases involving Asian individuals. Consequently, our approach enhances rapid screening capabilities, which can inform early-stage investigations while complementing subsequent, more comprehensive genetic analyses.

## 1 Introduction

Rapid ancestry inference from DNA evidence recovered at crime scenes provides crucial initial guidance for forensic investigations, particularly during the early phases when time constraints render comprehensive genetic analyses impractical. In these scenarios, Y-chromosome short tandem repeats (Y-STRs) offer a key advantage: their extraction and analysis protocols are considerably faster than those for more comprehensive genetic marker panels (Butler, 2011).

Northeast Asia is one of the world’s most genetically intricate and geopolitically dynamic regions. This area, which includes Korea, China, and Japan as its principal nations, is characterized by populations that possess unique genetic profiles yet share deep interconnections forged through millennia of migration, admixture, and cultural exchange (Horai et al., 1996; Du et al., 1997; Zhang et al., 2007; Bai et al., 2016; Yang, 2022). The substantial genetic overlap among these populations presents a challenge for linking genetic data to national origin, particularly as commercial ancestry panels often classify all Northeast Asians as a single homogeneous group (Li et al., 2016; Wang et al., 2019).

A significant limitation in current forensic practice is the failure of most commercial Ancestry Informative Marker (AIM) panels to effectively differentiate among Northeast Asian populations, often treating them as a single genetic entity. This overgeneralization hinders investigative efforts in regions where distinguishing between these populations could provide crucial leads. Although comprehensive genetic analyses using multiple marker types (e.g., autosomal STRs, SNPs, and mtDNA) yield the most definitive results, they require substantial time and resources that are often unavailable during the critical initial hours of an investigation (Alladio et al., 2022).

Y-STRs have emerged as valuable tools for initial ancestry screening, offering faster processing times than more comprehensive genetic analyses (Nazir et al., 2016). Their exclusively paternal inheritance allows for the identification of paternal lineages and provides population-specific signals that can guide early-stage investigations (Jeong et al., 2018; Mohapatra et al., 2019). These characteristics make Y-STR markers particularly suitable as a rapid initial screening tool for ancestry assessment in time-sensitive scenarios.

Previous studies have effectively utilized Y-STR haplotype and haplogroup distributions to examine global population diversity and identify patterns of genetic variation across geographical regions (Kayser et al., 2003; Bai et al., 2016; Nazir et al., 2016). Research in Northeast Asia has highlighted the application of Y-STR data in various contexts, including the documentation of novel mutations in Korean populations, sequence analyses of Japanese genetic profiles, comparative studies of genetic diversity between Tibetan and Han populations, and the identification of distinctive genetic signatures within Hakka communities (Horai et al., 1996; Hara et al., 2007; Bai et al., 2016; Jung et al., 2016; Wang et al., 2019; Watahiki et al., 2019). However, much of the existing research relies heavily on haplogroup-based approaches conducted at the group level. While beneficial for general phylogenetic studies, these methods may lack the resolution required for the rapid, individual-level classification needed in forensic contexts.

Traditional statistical methods, including haplotype frequency estimation and Analysis of Molecular Variance (AMOVA), have been used to measure genetic variation. However, for populations in regions such as Northeast Asia that exhibit limited genetic differentiation and extensive historical admixture, the efficacy of these methods may be limited (Watahiki et al., 2019; Cao et al., 2022; Li et al., 2023). Machine learning and deep learning can extract detailed patterns from Y-STR data, offering an alternative approach for initial ancestry inference.

This study presents a machine learning method to classify individuals into East Asian populations using Y-STR profiles. The method is designed for rapid initial screening in forensic applications. The framework uses a one-vs-rest (OvR) strategy, where a separate classification model is trained for each population to estimate the probability that a sample belongs to that group. The OvR approach is well-established in machine learning, and its application is suitable here given the genetic characteristics of Northeast Asian populations (Wang et al., 2019; Cao et al., 2022; Li et al., 2023).

This study’s findings address a limitation of commercial ancestry panels by offering a tool for initial ancestry inference in cases involving individuals of Asian descent. By providing preliminary ancestry information during the early phase of an investigation, this approach can help manage the time gap between the need for immediate leads and the completion of comprehensive genetic analyses. The research contributes to forensic methods for differentiating Asian populations and provides a basis for initial screening in criminal investigations.

## 2 Materials and methods

### 2.1 Y-STR sample data acquisition

In this study, Y-STR data from individuals in South Korea, China, Japan, Mongolia, and Kyrgyzstan were analyzed using the PowerPlex^®^ Y System Kit. The marker panel included twenty male-specific loci: DYS19, the multicopy marker DYS385ab, DYS389I, DYS389II, the DYS390–DYS393 block, and the single-copy loci DYS437, DYS438, DYS439, DYS448, DYS487, DYS533, DYS570, DYS576, and DYS635, along with YGATAH4 (Song et al., 2019; Li et al., 2020). This set of Y-STRs is frequently used for initial forensic screening due to rapid processing times and established protocols (Jung et al., 2016; Mohapatra et al., 2019).

Data were compiled from publicly available sources to obviate ethical concerns associated with new human subject participation. Y-STR profiles for Han Chinese, Korean, and Japanese populations were initially sourced from the YHRD online database (Fu et al., 2023; Song et al., 2023). Data for ethnic minorities in China (Hui and Yi) and for Mongolian and Kyrgyz populations were subsequently obtained from scholarly literature published since 2010 (Fu et al., 2023; Song et al., 2023). The final dataset included the Han Chinese population, the Hui and Yi ethnic minorities from China, and populations from Mongolia, Korea, Japan, and Kyrgyzstan.

**FIGURE 1:**
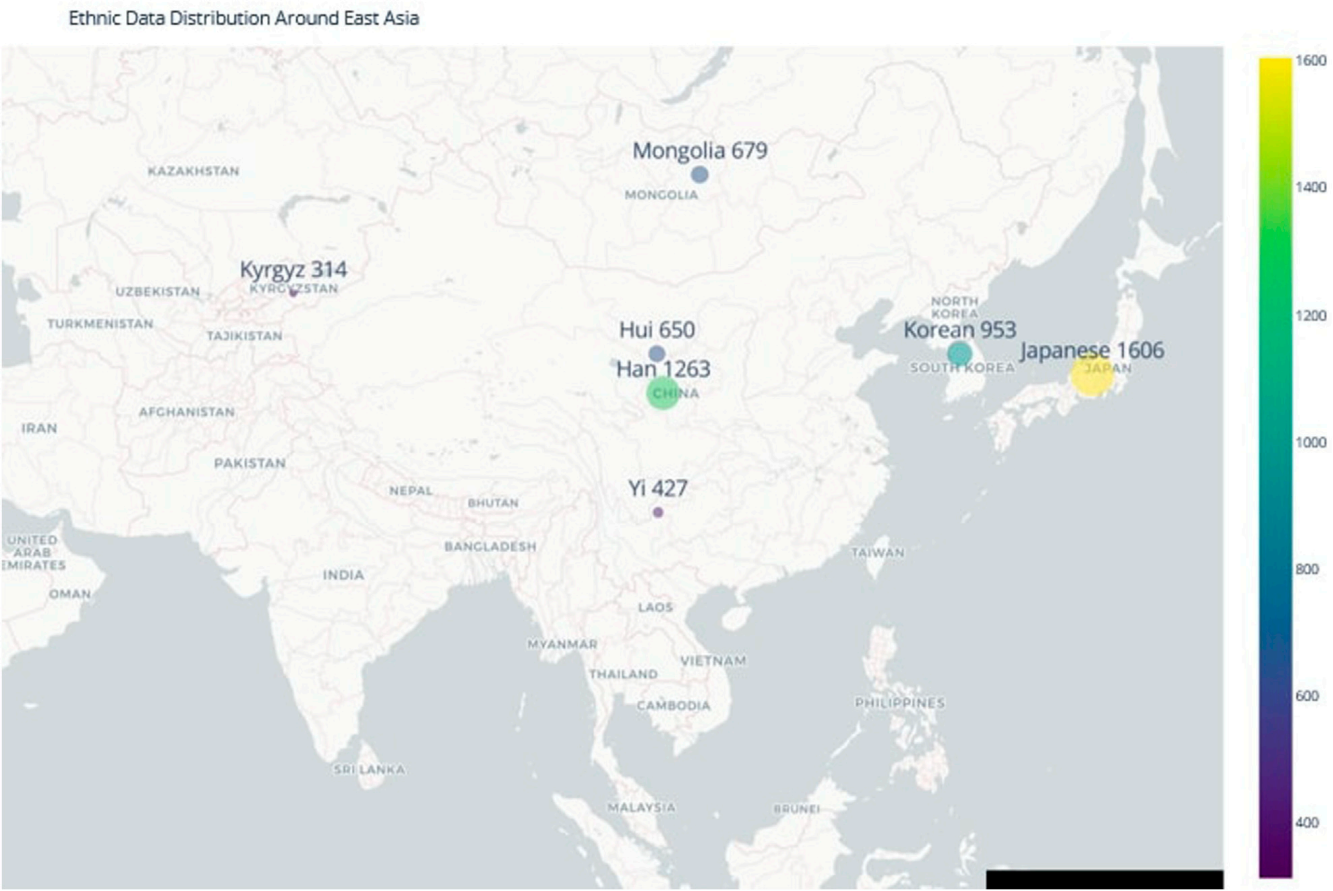
Geographic Diversity of Ethnic Populations in Northeast Asia. The map illustrates the geographical distribution of the populations included in this study, highlighting the complex population structure and migration patterns that have contributed to the genetic diversity of the region. Understanding this distribution is essential for accurate forensic ancestry inference in time-sensitive investigations.

The YHRD provides standardized and geographically diverse data, ensuring a consistent and reliable basis for cross-population comparisons (Su et al., 1999; Zhang et al., 2007; Ngamphiw et al., 2011; Lee et al., 2018; GenomeAsia 100K Project, 2019; Pan and Xu, 2020; Yang, 2022). This dataset was supplemented with data published in the late 2010s for Han, Kyrgyz, Mongolian, and Hui populations (Fu et al., 2016; Gao et al., 2016; Nazir et al., 2016; Jiang et al., 2017; Li et al., 2020). Sample sizes are detailed in Table 1, and the geographical distribution of the populations is shown in Figure 1.

**TABLE 1:** Sample distribution of Y-STR marker database.

Nation	Ethnic group	Database (N)
China	Han	839
	Hui	333
	Yi	273
Korea	Korean	520
Kyrgyzstan	Kyrgyz	220
Mongolia	Mongolia	443
Japan	Japanese	960

### 2.2 Designing ML models for ethnic identification

#### 2.2.1 Preprocessing for ethnic identification

The Y-STR data were preprocessed for analysis through data standardization, quality control, and feature transformation.

First, data from multiple sources were harmonized for compatibility with machine learning algorithms. As part of quality control, records with missing values or non-standard data formats were filtered to identify anomalies and inconsistencies in Y-STR loci distribution across populations. Y-STR profiles with empty values for any marker were excluded to maintain data integrity, as missing data can introduce bias or reduce model efficiency. This filtering step removed entries that could otherwise lead to unreliable model training outcomes (Pedregosa et al., 2011).

Second, feature transformation was performed to convert the Y-STR data into a suitable format for machine learning. The input data were derived from genotype information at 20 Y-STR loci, with multi-copy markers like DYS385ab treated as separate loci. All unique alleles at each locus, including microvariants (e.g., 13.2), were identified and treated as distinct categories. Subsequently, one-hot encoding was applied to transform each allele into an independent binary feature. This process generated a final input matrix of 245 binary features, where each feature indicates the presence (1) or absence (0) of a specific allele at a given locus. For the target variable, ethnic labels were converted from string to integer format using a LabelEncoder (Pedregosa et al., 2011).

Finally, the dataset was partitioned into training and test subsets at an 80:20 ratio. To mitigate class imbalance, downsampling was applied to match the sample size of the smallest population group (Kyrgyz). This approach was intended to prevent bias in the individual OvR models and ensure equal representation of all populations during model training (Tougui et al., 2021; Sun et al., 2022).

#### 2.2.2 ML classification models for identification of ethnicities in OvR based individuals

A one-vs-rest (OvR) methodology was used to address the multi-class classification task. This approach decomposes the problem by training a separate binary classifier for each of the seven populations (Wu et al., 2005). Such a strategy is advantageous in contexts with complex class interactions, as it allows each model to learn the specific decision boundary for a single population against all others.

For each population, an independent binary classifier was constructed. The model was trained using an OvR configuration where the target population was treated as the positive class and the remaining six populations were combined into a single negative class. The final multi-class prediction for a given sample was obtained by aggregating the outputs from all seven classifiers. Three algorithms were evaluated for use as the binary classifiers: Logistic Regression, Random Forest, and XGBoost (Rigatti, 2017; Li et al., 2019).

To optimize performance, hyperparameter tuning for each classifier was conducted using **Bayesian optimization** with stratified k-fold cross-validation. Unlike random search, this approach iteratively builds a probabilistic model of the objective function to select the most promising hyperparameters for evaluation, enabling a more efficient search of the parameter space (Tougui et al., 2021). This process also served to assess generalization and mitigate overfitting risks. Following tuning, the final OvR classifiers were retrained on the entire training dataset with their respective optimal hyperparameters.

For supplementary sensitivity analysis, a transformer named SelectOneFeaturePerMarker was used to reduce dimensionality. This method selected the single allele feature per locus with the highest mutual information relative to the target variable. However, all primary results reported in this study were generated using the full 245-feature set. The reduced 20-feature set was used only to evaluate model robustness under feature constraints. As noted in the Limitations section, this dimensionality reduction may discard information useful for discriminating between closely related populations.

#### 2.2.3 Platt Scaling based probability calibration

Machine learning models frequently encounter issues related to prediction error accumulation or exhibit overfitting/overconfidence bias toward specific classes in their probability outputs. To address these challenges, we applied Platt Scaling, a probability calibration methodology that enhances the reliability of model-generated predictions (Böken, 2021).

Platt scaling transforms raw scores (or probabilities) s∈[0,1] produced by classifiers through application of a logistic function (as shown in Equation 1):

$$P\left(y=1|s\right)=\frac{1}{1+\exp\left(As+B\right)}$$ (1)

In this equation, parameters A and B are estimated through cross-validation processes, while s represents the classifier’s default probability or logit score. The calibrated probabilities resulting from Platt Scaling implementation provide adjustments to compensate for probability overestimation or underestimation in model outputs (Böken, 2021). Having established the comprehensive network of associated parameters, this calibration process improved the accuracy of probability values subsequently utilized in attention meta-learning phases.

Since probability values in predictions often exhibit steepness and limited variance, we incorporated Platt Scaling to preserve these values for future calculations. This approach proved advantageous considering that reliable probability estimates are essential for subsequent attention-based meta-learning procedures (Park et al., 2020).

#### 2.2.4 Attention-based meta learning

We introduce an efficient, powerful multi-class meta-learner founded on attention mechanisms that consolidates outputs from multiple OvR binary classifiers. With seven ethnic groups in our study, each sample generated predictions from seven distinct binary classifiers, calibrated using Platt Scaling. These predictions follow a structured output format (sample × seven classifiers × two classes). While averaging classifier predictions might seem intuitive, this approach implicitly assumes equal importance across all classifiers in most scenarios—an assumption that proves inaccurate when considering varying difficulties in distinguishing between ethnic groups or differential sensitivities of specific genetic markers (Park et al., 2020).

Our Attention mechanism implementation follows a three-dimensional process:

First, we generate attention weights for probability outputs from each classifier. At this level, we employ weighted summation, with weights reflecting classifier accuracy for specific ethnic groups, determined through training performance metrics and mutual information assessments (Park et al., 2020).

Second, we implemented a weighted sum calculation to combine attention weights with classifier output probabilities according to Equation 2:

$$\mathrm{fi}nal\text{\_}prediction=\Sigma \left({attention\text{\_}weight}_{i}\times {classi\mathrm{fi}er\text{\_}probability}_{i}\right)$$ (2)

Finally, these weighted predictions underwent processing through a streamlined deep neural network comprising fully connected layers implemented via PyTorch (Park et al., 2020). We obtained final ethnic label predictions by passing outputs through this network, optimized using cross-entropy and backpropagation techniques.

This attention-based methodology offers numerous advantages over conventional aggregation approaches (Park et al., 2020). It enables more targeted management of individual models and enhances overall discriminative performance by ensuring each classifier’s contribution is dynamically adjusted based on genetic input features. This facilitates improved handling of scenarios where certain ethnic distinctions appear ambiguous or genetic features demonstrate varying discriminatory effectiveness. Through neural network learning processes, complex patterns in weighted predictions can be recognized, enabling differentiation between closely related ethnic groups. This approach heightens ethnic classification sensitivity while maintaining responsiveness to subtle genetic differences between groups, thereby improving result generalizability (Park et al., 2020; Barash et al., 2023).

Our application of attention mechanisms for classifier selection enabled the system to learn the relative importance of different classifiers in ethnic identification processes (Park et al., 2020). This proves particularly valuable when evaluating complex genetic relationships between closely related populations, where fixed-weight approaches often fail to distinguish subtle yet significant variations of interest.

## 3 Results

This section presents a comparative analysis of two machine learning architectures for Y-STR-based multi-population classification: a One-vs-Rest (OvR) model with an attention mechanism and a One-vs-One (OvO) stacking model. The analysis includes the performance of both models, their classification reports, confusion matrices, and the key genetic markers (alleles) identified by each. Based on these results, the OvR architecture was selected as the final model due to its balance of predictive accuracy and computational efficiency.

### 3.1 Comparative performance of OvR and OvO architectures

The overall performance of the two pipelines was first evaluated. The OvR model requires training seven binary classifiers (one for each population), whereas the OvO model requires training 21 classifiers.

Table 2 presents the overall performance metrics for both models, averaged across all cross-validation folds. The OvO model achieved slightly higher accuracy (+1.52%) and F1-score (+0.92%). However, this marginal performance gain was associated with a threefold increase in the number of classifiers, resulting in a greater computational load. Given the requirements of forensic and population genetics research, the trade-off between a minor accuracy improvement and a substantial increase in complexity is a critical consideration. Therefore, the OvR model was selected as the preferred architecture.

**TABLE 2:** Overall performance and complexity comparison of the two architectures.

Pipeline architecture	Accuracy	Precision	Recall	F1-score	No. Of classifiers
OvR with Attention	0.8031	0.8085	0.8031	0.8011	**7**
OvO Stacking Model	0.8183	0.8357	0.8183	0.8103	21

### 3.2 Detailed classification performance by population

The detailed classification reports for each population are shown in Table 3. The OvO model showed an improvement in recall for the Han (0.86–0.95) and Korean (0.74–0.83) populations, suggesting its pairwise approach may better capture the nuances of these groups. Conversely, the OvR model showed higher precision for the Mongolian (0.93 vs. 0.91) and Yi (0.96 vs. 0.94) populations. Neither model resolved the challenge of classifying the Hui population, which exhibited low recall in both architectures. This suggests that the difficulty in classifying this group is likely due to the data’s genetic distribution rather than a limitation of a specific model architecture (Li et al., 2020).

**TABLE 3:** Detailed classification performance metrics by population for both models.

Population	OvR with attention			OvO stacking model		
	Precision	Recall	F1-Score	Precision	Recall	F1-Score
Han	0.70	0.86	0.77	0.70	0.95	0.80
Hui	0.68	0.56	0.61	0.83	0.46	0.59
Japanese	0.93	0.94	0.94	0.96	0.92	0.94
Korean	0.77	0.74	0.75	0.77	0.83	0.80
Kyrgyz	0.93	0.95	0.94	0.90	0.98	0.94
Mongolia	0.93	0.74	0.83	0.91	0.77	0.83
Yi	0.93	0.80	0.87	0.94	0.80	0.86

### 3.3 Confusion matrix analysis

**FIGURE 2:**
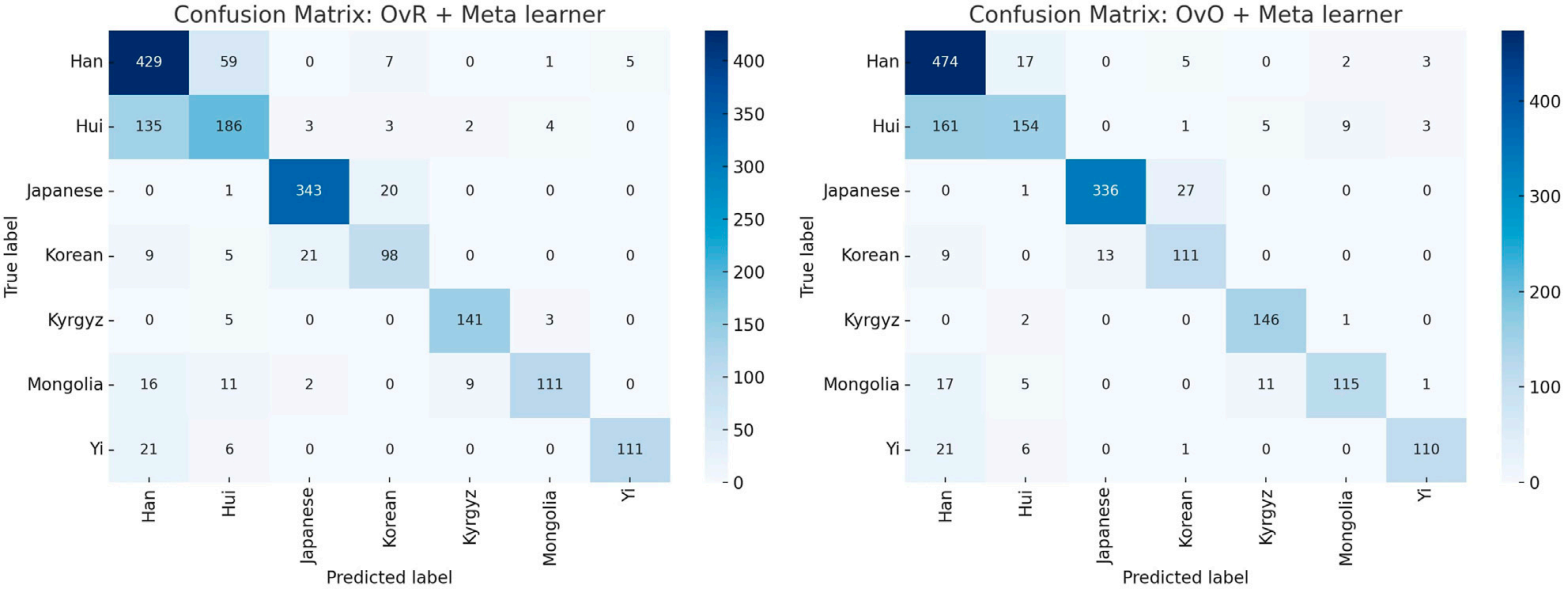
Comparison of confusion matrices for the final classification models using OvR + Meta learner (left) and OvO + Meta learner (right). The visualization highlights the strengths and weaknesses of each approach across different ethnic groups, showing consistent performance for Japanese and Korean populations, while indicating misclassification challenges for certain groups such as Hui and Han.

Figure 2 compares the confusion matrices of the two final classification models, providing a visual summary of their classification performance. Analysis of the confusion matrices _~_ between the Han and Hui populations (Table 4). A large proportion of Hui samples were misclassified as Han in both models. While the OvO model reduced the number of Han samples misclassified as Hui, it concurrently increased the misclassification of Hui as Han. This indicates that the more complex model did not resolve this issue, further supporting the selection of the more efficient OvR model. Misclassifications between the Japanese and Korean populations were also observed, though to a lesser extent, reflecting their known genetic proximity (Hara et al., 2007; Jung et al., 2016).

**TABLE 4:** Summary of key misclassifications from confusion matrices.

True population	Predicted As	OvR model errors (count)	OvO model errors (count)
Hui	Han	135	161
Han	Hui	59	17
Japanese	Korean	20	27
Korean	Japanese	21	13

These findings suggest that the data distributions for certain populations, particularly Hui and Han, overlap substantially. This complicates accurate classification regardless of model architecture and suggests a need for additional feature engineering or the inclusion of more discriminative genetic markers (Jin et al., 2021).

### 3.4 Pairwise OvO classification accuracy

To provide a more comprehensive representation of the OvO binary classification results, we report the pairwise accuracies across all ethnic groups. This matrix highlights population pairs that are relatively difficult to distinguish (e.g., Han–Hui) and those that are more readily separable (e.g., Japanese–Yi).

As presented in Table 5, the Han–Hui pair exhibits the lowest classification accuracy (0.787), which is consistent with known genetic overlap and admixture between these populations. In contrast, pairs such as Japanese–Yi and Han–Kyrgyz achieve near-perfect accuracies ( > 0.98), indicating clear genetic differentiation.

**TABLE 5:** Pairwise OvO binary classification accuracies among ethnic groups.

	Han	Hui	Japanese	Korean	Kyrgyz	Mongolian	Yi
Han	–	0.787	1.000	0.972	1.000	0.966	0.956
Hui	0.787	–	0.989	0.976	0.981	0.927	0.932
Japanese	1.000	0.989	–	0.928	1.000	0.998	1.000
Korean	0.972	0.976	0.928	–	1.000	0.993	0.974
Kyrgyz	1.000	0.981	1.000	1.000	–	0.856	1.000
Mongolian	0.966	0.927	0.998	0.993	0.856	–	0.969
Yi	0.956	0.932	1.000	0.974	1.000	0.969	–

### 3.5 Feature importance in the selected OvR model

An advantage of the selected OvR architecture is the interpretability of its individual binary classifiers. By analyzing the feature importance scores from each classifier, the specific Y-STR alleles that are most discriminative for each population were identified. Table 6 lists the most predictive alleles for several populations.

**TABLE 6:** Most discriminative alleles for key populations identified by the OvR model.

Population	Most discriminative allele (Marker_Allele)	Importance score
Kyrgyz	DYS439_10	0.976
Japanese	DYS439_12	0.929
Han	DYS389I_12	0.907
Yi	DYS448_17	0.897
Korean	DYS19_16	0.879

For example, the allele DYS439_10 (Score: 0.976) is a strong predictor for the Kyrgyz population, while DYS439_12 (Score: 0.929) is characteristic of the Japanese population. These high-scoring, population-specific alleles validate the model’s ability to learn biologically relevant patterns and provide interpretability for forensic applications.

In summary, the selected OvR with Attention model achieved an overall accuracy of 80.31% and a weighted F1-score of 0.8011. While it demonstrated high performance for genetically distinct populations such as the Japanese, Korean, and Kyrgyz, its primary limitation was the classification of the genetically similar Han and Hui populations. The model’s feature importance analysis successfully identified key discriminative alleles for most populations, providing a degree of interpretability. These results indicate that the OvR architecture provides a computationally efficient and reasonably accurate framework for initial ancestry screening, though challenges remain for differentiating closely related groups with the current Y-STR marker set.

## 4 Discussion

This study presents a framework that integrates tree-based ensemble models with a One-vs-Rest (OvR) classification strategy for rapid initial ancestry assessment from Y-STR data in forensic contexts. The focus on Y-STR markers, despite the utility of other markers like mtDNA or AIM-SNPs, was intentional. This decision was based on three considerations: (1) the potential of STR-only data for ancestry prediction is a relatively unexplored research area; (2) the paternal inheritance of Y-STRs makes them robust for tracing paternal lineages, which can be less affected by recent admixture than autosomal markers; and (3) the use of core loci from common commercial kits ensures the framework’s applicability to routinely generated forensic data.

Consistent with this focus on rapid screening, a direct performance comparison with AIM-SNP panels or Y-haplogroup tools was not conducted. Such a comparison was precluded by differences in data availability and because the primary advantage of this Y-STR framework is its operational speed. It is designed to provide preliminary guidance within hours, a critical requirement in early-stage investigations that SNP-based or sequencing analyses typically cannot meet.

The developed framework achieved an overall accuracy of 81% using only Y-STR data, a competitive performance level given the low genetic differentiation among Northeast Asian populations. The value of this framework lies not in providing definitive evidence for suspect identification, but in its function as a supplementary tool to guide initial investigative efforts. For example, when a crime scene profile has no database match, the model can offer a probabilistic assessment (e.g., “75% probability of Korean origin, 15% of Han Chinese origin”) to help prioritize resources.

To further assess the genetic distinguishability between populations, pairwise One-vs-One (OvO) classification experiments were also conducted. The results indicated that Japanese and Korean populations were more accurately classified compared to continental groups like the Han Chinese, which aligns with previous findings of their distinct genetic profiles. Conversely, lower accuracy was observed between geographically proximate or historically interconnected groups, such as the Han Chinese and Hui, suggesting genetic admixture or shared ancestry.

Finally, to address the interpretability of the attention mechanism, we analyzed its weighting process. The attention meta-learner dynamically assigns higher weights to the expert models most relevant to a given input. The analysis showed that for an input from a specific population, the corresponding expert model consistently received a high weight. Furthermore, the genetic loci deemed important by these highly weighted models were consistent with those reported in the literature as discriminative for that population, such as specific alleles at DYS390 and DYS576 for Korean and Japanese populations (Hara et al., 2007; Jung et al., 2016). This indicates that the model’s dynamic weighting is based on biologically relevant patterns, enhancing its transparency.

## 5 Limitations

Although the proposed Y-STR–based One-vs-Rest (OvR) attention-ensemble framework demonstrates competitive performance for rapid ancestry inference in Northeast Asian populations, several limitations should be acknowledged.

**FIGURE 3:**
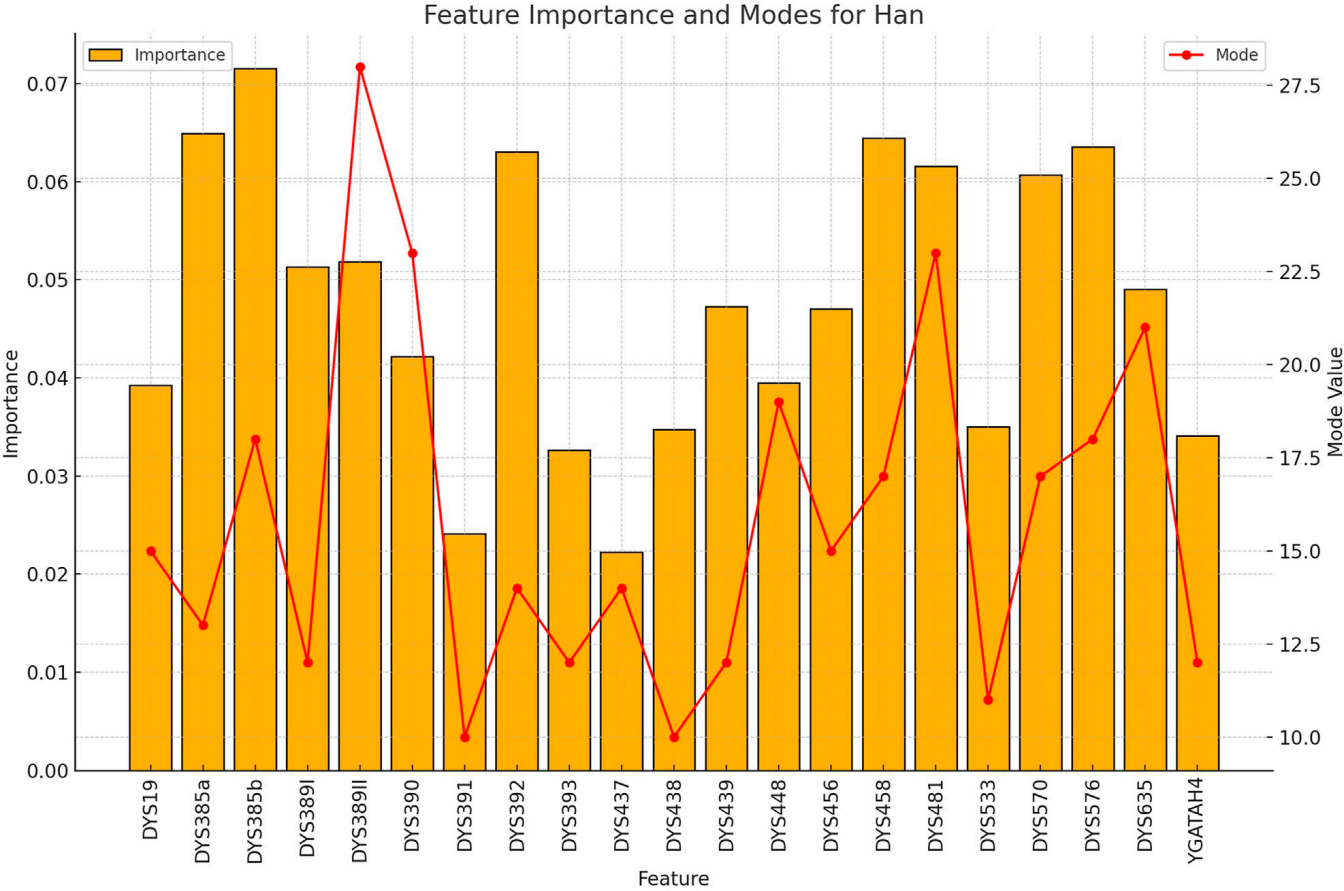
The importance and representative allele of each Y-STR loci marker of Han Population. The relative importance values (left y-axis) and associated allele values (right y-axis) demonstrate which genetic markers provide the most discriminative power for identifying Han individuals in a rapid screening context.

**FIGURE 4:**
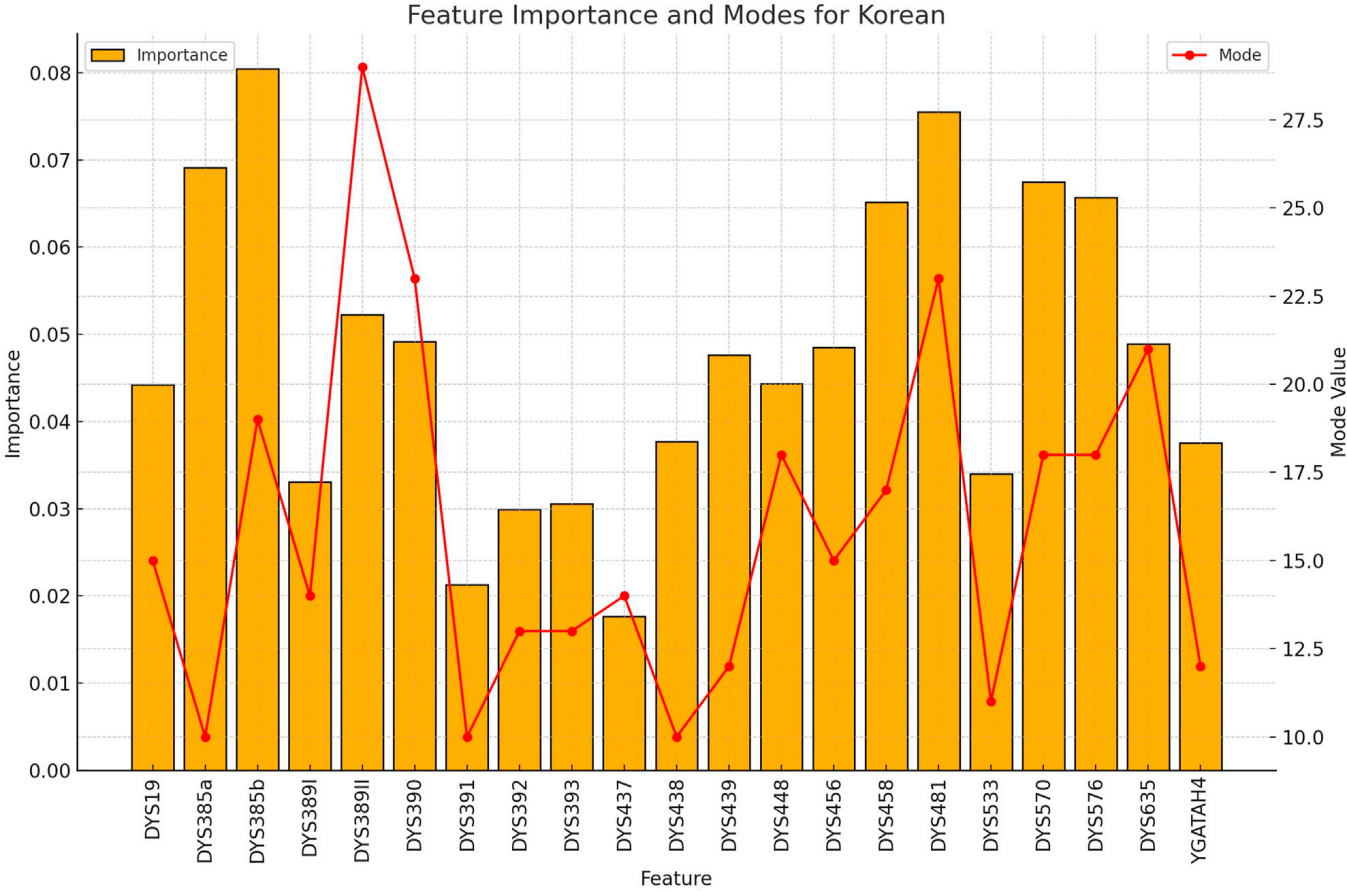
The importance and representative allele of each Y-STR loci marker of Korean Population. The consistent pattern of highly discriminative markers explains the exceptional performance of the model for Korean samples, supporting the value of Y-STR markers for rapid nationality inference.

**FIGURE 5:**
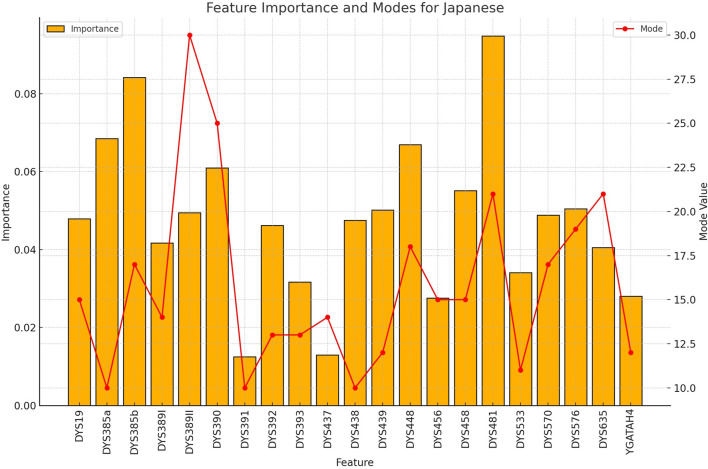
The importance and representative allele of each Y-STR loci marker of Japanese Population. The distinctive pattern observed here contributes to the perfect classification accuracy achieved for Japanese samples, demonstrating the potential of Y-STR markers for certain Northeast Asian populations.

**FIGURE 6:**
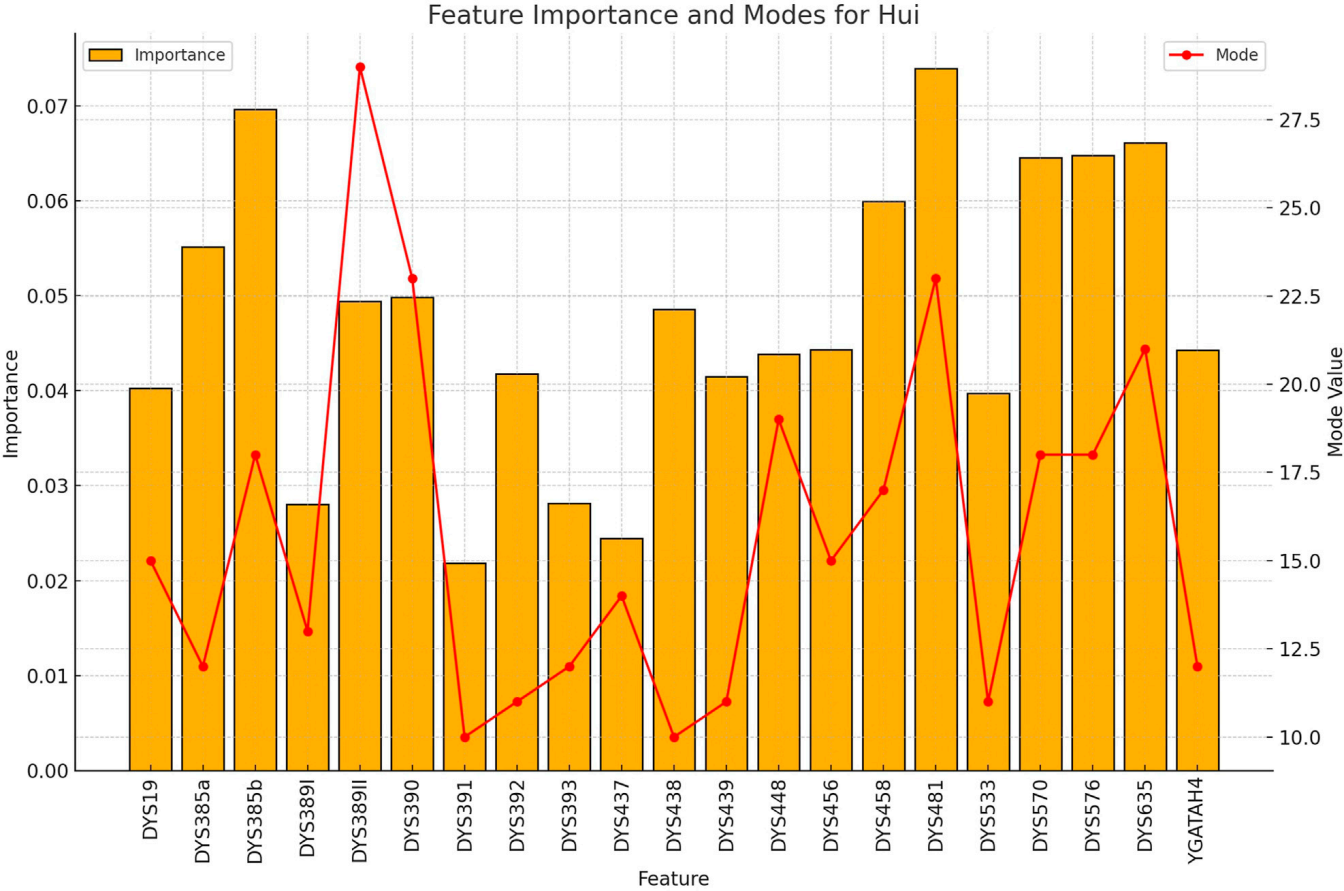
The importance and representative allele of each Y-STR loci marker of Hui Population. The less distinctive pattern observed here correlates with the lower classification accuracy for this ethnic group, highlighting areas where future refinement may improve performance.

**FIGURE 7:**
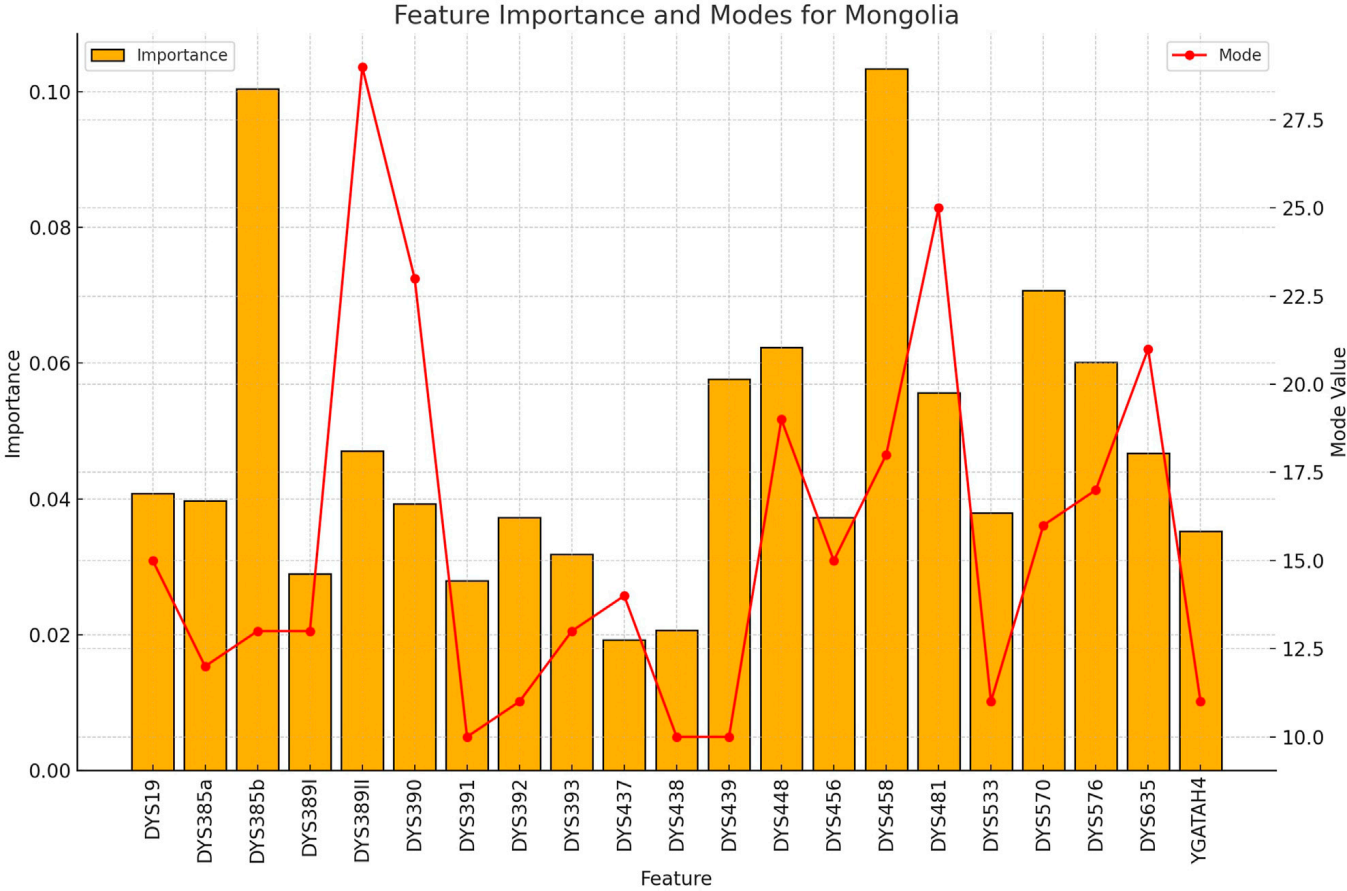
The importance and representative allele of each Y-STR loci marker of Mongolia Population. This visualization helps explain the moderate classification performance for Mongolian samples, with certain markers showing distinctive patterns while others overlap with neighboring populations.

**FIGURE 8:**
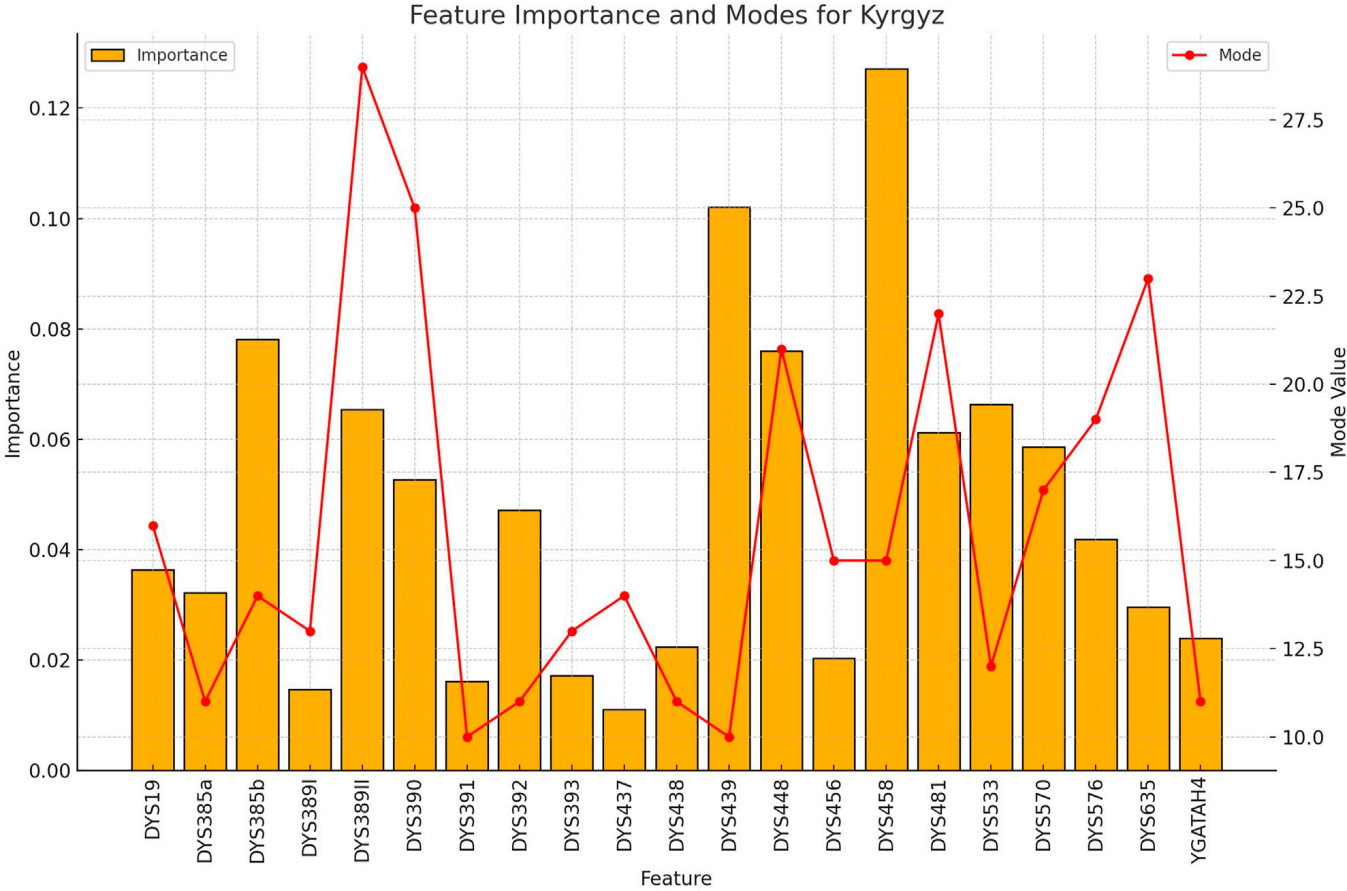
The importance and representative allele of each Y-STR loci marker of Kyrgyz Population. The consistency of key markers with high importance values contributed to the robust performance in identifying this population group, despite having the smallest sample size.

**FIGURE 9:**
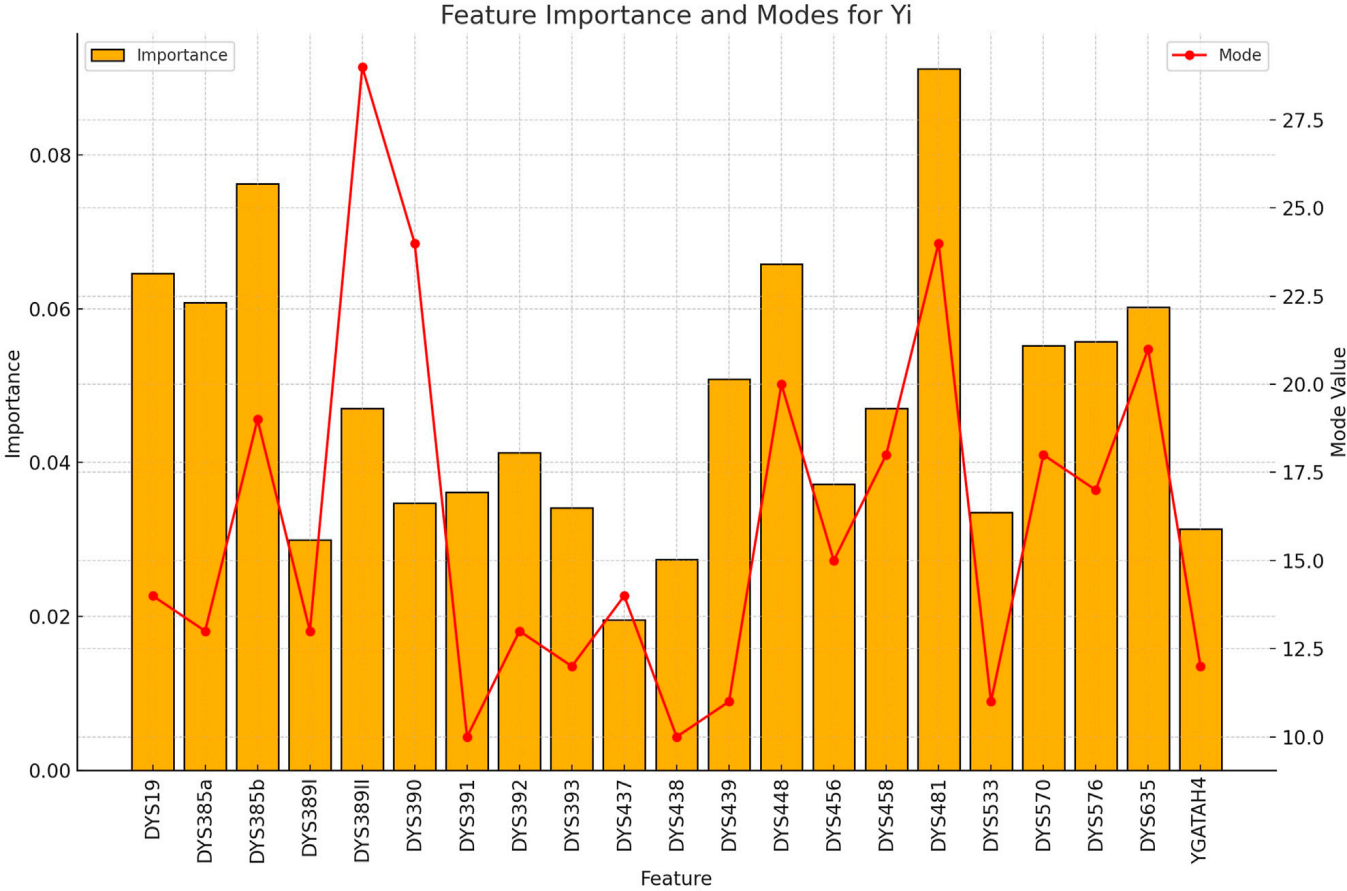
The importance and representative allele of each Y-STR loci marker of Yi Population. The feature importance pattern reveals which markers are most valuable for distinguishing the Yi ethnic group from others in rapid forensic screening applications.

1. Sample Size and Class Imbalance: The dataset exhibits a pronounced imbalance across ethnic groups, with certain populations (e.g., Hui and Mongolian) being underrepresented. This imbalance, coupled with the small sample sizes for minority groups, likely contributed to lower performance for these populations (e.g., Hui: F1 ≈ 0.53; Mongolian: recall ≈ 0.50) and increased susceptibility to overfitting. Although downsampling was applied to balance the training data, this method also reduced the effective training size for majority classes.
2. Marker Set Constraints: The study relied exclusively on 20 Y-STR loci, selected from the overlapping core of commercial forensic kits to maximize real-world applicability. While Y-STRs enable rapid paternal lineage inference, they do not capture maternal ancestry and offer limited resolution in mixed-DNA scenarios. The restricted marker set also constrains the discriminative capacity for closely related or admixed populations.
3. Representation of Mixed Ancestry: Because Y-STRs reflect only paternal lineage, the framework is not optimized for detecting recent admixture involving maternal contributions or complex multi-lineage backgrounds. In such cases, predictions may predominantly represent paternal origin, potentially overlooking other ancestral components.
4. Feature Selection Trade-offs: The optional “SelectOneFeaturePerMarker” transformer, used in supplementary sensitivity analyses, reduces dimensionality by retaining only the allele with the highest mutual information per locus. While effective in mitigating overfitting in small datasets, this approach discards multi-allelic information, which may diminish discriminatory power for genetically similar populations. The primary results were obtained using the full 245-dimensional one-hot feature set; however, future work should explore top- k allele selection or embedding-based encodings to better preserve allelic diversity.
5. Dependency on Probability Calibration: The attention-based meta-learner relies on well-calibrated probability estimates from its base classifiers. Initial evaluations revealed an overconfidence bias in raw model outputs, particularly for minority groups, necessitating Platt scaling. While calibration improved the reliability of probability magnitudes without substantially affecting accuracy, it introduces an additional processing step and assumes the stability of calibration across datasets.
6. Interpretability of the Attention Mechanism: Although per-population marker–allele importance plots (Figures 3–9) support the biological plausibility of the attention weights, the meta-learner’s dynamic weighting remains a data-driven process rather than a direct causal mapping. This “black-box” characteristic may limit forensic transparency, particularly in legal contexts requiring fully interpretable decision rules.
7. Generalizability to External Data: The reported performance metrics are based on cross-validation within a specific dataset compiled from YHRD and published literature. Variations in genotyping kits, allele binning, or population structure in external datasets could diminish accuracy. The highest reliability in the present study was observed for Japanese and Korean populations; extending applicability to other groups will require larger, more balanced, and geographically diverse reference datasets.
8. Operational Scope in Forensic Contexts: While the framework can provide rapid, probabilistic ancestry assessments to inform early investigative decisions, it should not be regarded as definitive evidence of an individual’s ethnicity. Misclassification—particularly for minority groups—may bias investigative focus if results are not interpreted alongside other lines of evidence.

## 6 Conclusion

This study demonstrates that a machine learning framework combining a One-vs-Rest (OvR) strategy with an attention-based meta-learner can classify individuals into Northeast Asian populations using only Y-STR data. The final model achieved an overall accuracy of 81%, indicating its utility for rapid initial ancestry screening in forensic contexts. The approach offers a method to differentiate among Northeast Asian populations often aggregated into a single category by commercial ancestry panels, thereby providing preliminary guidance in the early stages of an investigation.

Methodological components such as the SelectOneFeaturePerMarker transformer for dimensionality reduction and Platt scaling for probability calibration contributed to the model’s development and reliability. While the framework performed well for genetically distinct populations, its performance was limited for genetically similar groups like the Han and Hui, primarily due to data imbalance and overlapping Y-STR profiles. These results highlight the challenges that persist in classifying closely related populations.

To address these limitations, future work could focus on several areas. Exploring alternative attention mechanisms or model architectures, such as neural networks, may improve performance. More advanced feature engineering could also better capture the information within Y-STR markers. Additionally, expanding the dataset to include more samples from underrepresented groups and developing frameworks to integrate Y-STR data with other genetic markers would be valuable next steps.

In conclusion, this research presents a computationally efficient framework for initial ancestry screening of Northeast Asian populations. By addressing data imbalance and leveraging an interpretable model architecture, this work provides a practical tool for forensic applications and a basis for future research in high-resolution ancestry inference.

## Data Availability

Publicly available datasets were analyzed in this study. This data can be found here: The datasets analyzed in this study are publicly available through the YHRD database (https://yhrd.org), as well as through previously published literature cited in the manuscript.
